# Self-generated sounds of locomotion and ventilation and the evolution of human rhythmic abilities

**DOI:** 10.1007/s10071-013-0678-z

**Published:** 2013-08-30

**Authors:** Matz Larsson

**Affiliations:** 1 The Cardiology Clinic, Örebro University Hospital, 701 85 Örebro, Sweden; 2 The Respiratory Clinic, Örebro University Hospital, 701 85 Örebro, Sweden; 3 Institute of Environmental Medicine, Karolinska Institutet, Stockholm, Sweden

**Keywords:** The origins of music, Vocal learning, Primate, Entrainment, Auditory masking, Collective behavior

## Abstract

It has been suggested that the basic building blocks of music mimic sounds of moving humans, and because the brain was primed to exploit such sounds, they eventually became incorporated in human culture. However, that raises further questions. Why do genetically close, culturally well-developed apes lack musical abilities? Did our switch to bipedalism influence the origins of music? Four hypotheses are raised: (1) Human locomotion and ventilation can mask critical sounds in the environment. (2) Synchronization of locomotion reduces that problem. (3) Predictable sounds of locomotion may stimulate the evolution of synchronized behavior. (4) Bipedal gait and the associated sounds of locomotion influenced the evolution of human rhythmic abilities. Theoretical models and research data suggest that noise of locomotion and ventilation may mask critical auditory information. People often synchronize steps subconsciously. Human locomotion is likely to produce more predictable sounds than those of non-human primates. Predictable locomotion sounds may have improved our capacity of entrainment to external rhythms and to feel the beat in music. A sense of rhythm could aid the brain in distinguishing among sounds arising from discrete sources and also help individuals to synchronize their movements with one another. Synchronization of group movement may improve perception by providing periods of relative silence and by facilitating auditory processing. The adaptive value of such skills to early ancestors may have been keener detection of prey or stalkers and enhanced communication. Bipedal walking may have influenced the development of entrainment in humans and thereby the evolution of rhythmic abilities.

## Introduction

Throughout human history, music has played a major role in all cultures, but the origins of music remain mysterious (Hauser and McDermott 2003). Some suggest that music evolved as a system to attract mates and to signal mate quality (Darwin 1871/1981; Miller 2000; Pinker 1997), and others suggest that music functions to coordinate coalitions (Hagen and Bryant 2003). Pinker proposed that music may be a fortuitous side effect of diverse perceptual and cognitive mechanisms that serve other functions (Pinker 1997). Clarke (2005) stated that music and language exemplify how culture and biology have become integrated in complex ways. It has been proposed by Chater et al. (2009), Darwin (1871/1981), and Wilson (2011, p. 225–235) that the development of language from its underlying processing mechanisms arose with language evolving to fit the human brain, rather than the reverse, and an analogous situation has been proposed for music (Clarke 2005; Pinker 1997; Changizi 2011). However, the most advanced cultures known in animals, those of the chimpanzee and the bonobo (Wilson 2011), lack even rudimentary musical abilities (Jarvis 2007; Fitch 2006). Why and how did humans evolve musical abilities, despite the fact that their closest relatives, apes, are not vocal learners (Jarvis 2004) and cannot entrain to external rhythms (Fitch 2006)? Trevarthen (1999) proposed that the bipedal walk and its accompanying consciousness of body rhythms have implications for our internal timing system as well as for freeing the arms for communicative purposes. Changizi (2011) hypothesized that the human brain was harnessed by music because humans are adept at listening and interpreting the meaning of footsteps. Thus, he suggests that music evolved to mimic footsteps and sooner or later became incorporated in human culture. The idea that sense of rhythm is linked with footsteps is not new. Morgan (1893, p. 290) wrote, “I would suggest that the psychological basis of the sense of rhythm might be found in… the organic rhythms of our daily life. We cannot walk nor breathe except to rhythm; and if we watch a little child we should obtain abundant evidence of rhythmic movements”. Here, possible links between human walking and rhythmic abilities are further explored, focusing on incidental sounds and vibrations produced as a by-product of locomotion and respiration. The review raises the question whether predictability of such self-generated sounds may boost the evolution of entrainment to external rhythms, and whether that in turn may advance vocal learning abilities. Accordingly, a fundamental question is whether human locomotion is likely to produce more predictable sounds than those of non-human primates. Moreover, what was the primary adaptive value of entrainment to external rhythms in human ancestors? Could a sense of rhythm aid the brain in distinguishing among sounds arising from discrete sources and also help individuals to synchronize their movements with one another? The following hypotheses are raised: (1) Human locomotion and ventilation can mask critical sounds in the environment. (2) Synchronization of locomotion reduces such masking problems. (3) Highly predictable sounds of locomotion in a species stimulate the evolution of synchronized locomotion. 4) The evolutionary switch to bipedalism and the associated sounds of locomotion influenced the evolution of human rhythmic abilities.

Auditory masking, mechanisms that suppress self-generated sound, and sounds of locomotion and ventilation across the animal kingdom with focus on primate locomotion, and then the synchronization of movements in human and non-human primates are explored. Finally, hypotheses are raised with respect to how bipedal locomotion may have stimulated the evolution of human rhythmic and musical abilities.

## Masking

Auditory masking occurs when the perception of a sound is affected by the presence of another sound. Masking effects are particularly strong when the masker and the signal are of the same frequency and weaken as the signal frequency moves further away from the masker frequency (Gelfand 2004). When two sounds are of identical frequency, the listener cannot distinguish between them and they are perceived as one sound with the lower-amplitude sound masked by the louder. Masking of differing frequencies requires that the amplitude of the competing sound be greater in order to produce a masking effect. A masker may be simultaneous, as when a signal is made inaudible by a competing sound of equal duration, or it may precede (forward masking) or follow the signal (backward masking). The effectiveness of forward and backward masking attenuates exponentially from the onset or offset of the masker (Marler et al. 2002; Moore 2003, pp. 107–116). Learning reduces backward masking; the brain adapts to repetitive sequences of masking noise emitted soon after a signal and learns to discriminate between signal and masker, thus substantially increasing signal detection (Kidd and Feth 1982; Moore 2003, pp. 107–116). Moore (2003, p. 107) states that the adaptive value of this learning effect is poorly understood. The study of whether learning reduces the masking potential of repetitive self-generated sounds of locomotion is of interest. No doubt animal auditory systems have developed other mechanisms to reduce masking from self-generated sounds.

## Suppression of the perception of self-generated sounds

An animal’s locomotion, breathing, and vocalizations produce sounds that may stimulate its own auditory system. A possible consequence is excessive stimulation (sensory reafference) of the auditory system or masking of signals originating in the surroundings (von Holst and Mittelstaedt 1950). Sensory reafference in relation to vocalization has been studied (Greenlee et al. 2011; Hawco et al. 2009), while sounds associated with locomotion and ventilation have received little attention.

Healthy adults take around 10,000 steps each day (Tudor-Locke and Myers 2001; Bohannon 2007) and approximately 15 breaths per minute throughout life. How does the auditory system avoid overstimulation and discriminate locomotion and ventilation sounds from critical sounds in the environment? Sperry (1950) coined the term “corollary discharge” (CD) for motor-related signals that influence sensory processing. Crapse and Sommer (2008a) have suggested that adaptation processes to compensate for motor-related sensory problems, such as sensory reafference, are remarkably consistent among species. In general, such adaptation involves concurrent production of a motor command destined for an effector and a motor-command copy destined for a sensory structure functioning to minimize, eliminate, or compensate for the movement-related noise (Crapse and Sommer 2008a). In other words, nervous systems keep track of movement commands and inform the system’s sensory processing areas about forthcoming movements (Crapse and Sommer 2008b). At the lower-order level of reflex inhibition and sensory filtration, CD is a discriminatory mechanism that prevents maladaptive responses and sensory saturation by restricting or filtering information. Thus, CD serves as a guard intervening at points along a sensorimotor pathway to regulate the sensory information entering the system (Crapse and Sommer 2008a). Higher-order CD signaling involves sensory analysis, sensorimotor planning, and learning (Crapse and Sommer 2008b). Corollary discharge signaling may improve human capacity to perceive variations in the environment and discriminate them from self-generated sensory consequences (Cullen 2004). Sensory attenuation of the effects of self-generated action has been described (Blakemore et al. 1999; Shergill et al. 2003; Aliu et al. 2009; Tsakiris and Haggard 2003; Sato 2008). Martikainen et al. (2005) found that responses in the human auditory cortex were significantly weaker to self-triggered sounds. Baess et al. (2009) compared auditory middle latency responses (MLR) evoked by self-initiated click sounds to responses to externally initiated but otherwise identical sounds and found that MLRs were significantly attenuated in the self-initiated condition. A self-generated sensory episode is usually perceived as less powerful than a similar sensory episode generated externally (Blakemore et al. 1999; Sato 2008). However, Desantis et al. (2012) observed that the accuracy of discrimination did not significantly differ between these conditions, indicating that self-generation does not necessarily reduce the amount of perceptual information being processed. Although sounds of locomotion and ventilation are, by definition, self-generated and extremely common, studies of their impact on perception and behavior are scarce.

## Incidental sounds of locomotion and ventilation in the animal kingdom

### Invertebrates

The auditory receptors of crickets are located on their forelegs, and as a consequence, walking produces excitation of auditory receptors in the absence of sound and suppression of action potentials in response to sounds (Schildberger et al. 1988). Female crickets orienting to a male calling song pause frequently and change direction primarily during pauses (Murphey 1972; Bailey and Thomson 1977). There is evidence that orientation is less effective when the song is heard only during moves than when it is heard only during pauses (Weber et al. 1981). The tympanic membrane of grasshoppers is situated near air sacs in the tracheal system; therefore, it is deflected inward and outward during the respiratory cycle (Meyer and Elsner 1995; Meyer and Hedwig 1995). These slow movements change its auditory response properties and modulate the afferent activity. Ventilation thus distorts the perception of conspecific communication signals. Singing males of *Chorthippus biguttulus* may arrange their ventilatory and stridulatory activity in a manner that leaves “windows” open for listening (Meyer and Elsner 1995; Meyer and Hedwig 1995). Parasitoid wasp species that detect their prey using vibrations in the substrate spend a higher proportion of time motionless than species that use their ovipositors to probe for prey (Vet and Bakker 1985) suggesting that movement interferes with detection of prey movement (Kramer and McLaughlin 2001).

### Spiders

The synchronized and rhythmical activity of the social spider *Anelosimus eximius* (Araneae, Theridiidae) is likely to promote prey localization (Krafft and Pasquet 1991). Synchronization of movements with resting periods (respected by all in the group) creates “silent” periods, during which the spiders may assess and locate the struggling prey.

### Vertebrates

Pressure waves/water movements caused by an individual’s own locomotion or breathing might interfere with lateral line and electrosensory perception in fish (Russell 1968, 1974; Roberts and Russell 1972) and in *Xenopus laevis* (Russell 1971). Swimming fish larvae were shown to display reduced responsiveness to flow stimuli and were 40 % as likely to respond to flow signals as motionless larvae, implying sensory benefits of intermittent swimming cessation (Feitl et al. 2010). Mechanisms to decrease the masking potential of fish breathing have been described. An adaptive filter in the medullary nuclei cancels self-induced breathing noise in the electrosensory and lateral line systems of fish (Montgomery and Bodznick 1994). Second-order electrosensory neurons in elasmobranch fish and mechanosensory neurons in teleost fish have adapted to cancel the effects of stimuli that are tied with fish respiratory movements (Montgomery and Bodznick 1994). It has been suggested that the need to cope with auditory masking problems associated with incidental sounds of locomotion influenced the evolution of synchronized behavior in fish groups. It is likely that schooling fish produce overlapping and confusing acoustical signals, which may result in predator confusion (Larsson 2009, 2012b). Since synchronized locomotion in vertebrate ancestors may have had highly adaptive functions, the vertebrate brain may be pre-programmed to develop synchronized behavior in other ecological niches, e.g., birds flying in formation and surface diving dolphins (Larsson 2012a).

## Signaling functions of sounds of locomotion

The fact that incidental sound of locomotion may be a masker in many situations does not contradict the possibility that sound produced during locomotion may create essential signals. Wing beats of certain characteristics in drosophila (Bennet-Clark et al. 1980), mosquitoes (Gibson and Russell 2006), moths (Bailey 1991), and some bird species, e.g., the flappet lark (Payne 1973; Norberg 1991) and hummingbird (Hunter 2008) have been suggested to produce audible intersexual advertisements. Wingbeats of certain characteristics may serve as a predator alarm in the mourning dove (Coleman 2008) and the crested pigeon (Hingee and Magrath 2009). Locomotion-related sound and water movements seem to play a crucial role in communication in schooling fish (Pitcher et al. 1976).

### Locomotion sounds in primates

Apes show a wide range of locomotion behaviors, including brachiation, quadrumanous (four-handed) climbing, quadrupedal knuckle or fist walking, and regular short bouts of bipedal locomotion (Schmitt 2003). Little is known about the sounds they produce during locomotion. However, studies of primate locomotion may give an idea to what extent these sounds may be regular and predictable. The coordination of limb movements of non-human primates was reviewed by Stevens (2006). While most mammals use lateral sequence gaits in which a forelimb follows an ipsilateral hind limb during the stride cycle, primates have a tendency to utilize diagonal sequence gaits, i.e., the contralateral forelimb follows a given hind limb during the stride cycle. Primates demonstrate a high degree of flexibility in gait sequence pattern, which is likely to offer advantages for moving through discontinuous and unstable tree limbs (Stevens 2006). Primates moving in trees usually strive to maintain contact with at least one limb, resulting in little or no aerial phase (O’Neill 2012; Schmitt et al. 2006). The distance between limbs, and their degree of flexibility, is likely to vary, leading to the lack of regular limb sequences (Thorpe et al. 2009). Orangutans control excess sway by using irregular gait patterns and multiple support limbs (Thorpe et al. 2009). Due to the fragmented nature of forest canopies, arboreal animals must often cross large gaps between trees (Channon et al. 2011). During locomotion on ground, the stride length and walking speed of chacma baboons were reported to vary considerably (Sueur 2011). Many non-human primates use bipedal gait opportunistically, moving on flexed limbs, “bent-hip, bent knee”, which probably was the earliest form of bipedal gait in the hominids (Demes and O’Neill 2013). Capuchin monkeys, basically arboreal quadrupeds, come to the ground frequently and, especially in the context of transport and tool use, often use bipedal gait (Demes and O’Neill 2013). Although bipedal gait is not exclusive to humans, data from bearded capuchin monkeys and adult African apes indicate that the average proportion of bipedal gait is no more than 1–2 % of total locomotion (Duarte et al. 2012). Moreover, non-human primates’ bipedal gait differs distinctly from human walking in that primates do not use pendulum-like walking (Demes and O’Neill 2013).

Human walking shows long-term regularities (Dingwell and Cusumano 2010; Hausdorff et al. 1996). During unconstrained over-ground walking, stride time, stride length, and speed exhibit strong statistical consistency (Terrier et al. 2005). The first hominids habitually using an upright bipedal gait probably evolved in Africa five to six million years ago (Schmitt 2003). Human walking on a flat surface is combined with oscillating movements of the legs, arms, and head (Goldberger et al. 2000; Nessler and Gilliland 2009). Laboratory studies have suggested that the preferred cadence of walking is approximately 120 steps per minute (SPM), which has also been demonstrated during extended periods of unconstrained locomotor activity (MacDougall and Moore 2005). While data about the characteristics of non-human primates’ locomotion sounds are lacking, human locomotor sound has been thoroughly examined.

### Sounds in human bipedal locomotion

Humans and other species often stop and listen if they need to detect a faint sound or to make a fine auditory discrimination (Kramer and McLaughlin 2001). Locomotion typically creates audible sounds containing a number of qualitatively dissimilar acoustical events: isolated impulsive signals, sliding sounds, crushing sounds, and complex temporal patterns of overlapping impulsive signals (Visell et al. 2009). Other airborne or bone-conducted locomotion sounds produced by arm movements, irregularities in joints, or clothing movements may also be perceived by a walker. Walking conveys information about the properties of the sound source, and even without explicit training, listeners learn to draw conclusions based on the features of the sound (Visell et al. 2009), including such aspects as the gender (Giordano and Bresin 2006; Li et al. 1991), posture (Pastore et al. 2008) and emotions of a walker (Giordano and Bresin 2006), and properties of the ground surface (Giordano et al. 2012). Due to the lack of data concerning the frequency and intensity of the sounds of locomotion at ear level, I obtained some preliminary data of sounds generated by a man walking on a beach and in shallow water. Locomotion sound was recorded approximately 5 cm from the walker’s ear with a portable dB meter (Table 1). Walking on sand and gravel increased the sound level 24 dB LAeq above baseline (from 38 to 62 dB) and walking in shallow water 32 dB LAeq (from 34 to 66 dB).^1^ The sound level and masking potential of locomotion sound are likely to be variable and be influenced, e.g., by locomotion patterns, the size of the walker, the substrate, and the characteristics of the signal. Self-generated locomotion sounds are likely to have a potential to mask the analogous footsteps’ sounds produced by a nearby individual, since footsteps on similar ground are likely to generate sounds of a similar bandwidth. Self-generated locomotion sounds (the masker) will usually have higher amplitude than those of a second individual (the signal) since the former is produced nearer to the listener. In addition, walking will result in self-generated sound transmitted to the inner ear via the bones of the skull (Moore 2003, pp. 22–23), which is likely to contribute to their masking potential. In the simple trial cited here, frequencies of locomotion sounds overlapped substantially with speech, indicating the potential to mask vocal communication.

**Table 1:** Sound levels 5 cm from the right ear of a 182 cm man

Environment/condition	dB value expressed in (LAFmin) LAeq (LAFmax)	
	Stationary	Walking
Gravel/sand at beach	(28) 38 (52)	(30) 62 (71)
Water’s edge	(26) 34 (48)	(32) 66 (75)

	Stationary, no breathing	Stationary, breathing (freq. 1/s)
Silent room	(17) 19 (26)	(24) 53 (66)

Recorded with a dB meter

LAFmin = minimum sound level, dB(A), LAeq = equivalent continuous level (see fact square), LAFmax = maximum sound level, dB(A)

Walking and running are periodic activities, with a single period known as the gait cycle (GC). By definition, the GC begins when one foot comes into contact with the ground and ends when the same foot contacts the ground again (Novacheck 1998). Human walking rates are generally in the range of 75 and 125 SPM (Sabatier et al. 2008), corresponding to a GC of 0.8–0.5 s. The GC is comprised of stance and swing phases (Novacheck 1998). In walking, the two initial portions of the stance phase (initial contact and the loading response) normally produce more sound energy than other stance phase portions, although their combined duration is less than 10 % of the GC (Novacheck 1998). A walking sound is usually a sequence of isolated impact sounds generated by a temporally limited interaction between two objects (Visell et al. 2009). The foot and ground exert an equal and opposite force on one another, the “ground reaction force” (GRF) (Novacheck 1998), which is associated with the movement of the center of the mass of the individual (Galbrait and Barton 1970). It has been demonstrated in capuchin monkeys that GRFs are larger in bipedal gait than in quadrupedal locomotion (Demes and O’Neill 2013). In acoustics, the term GRF usually refers to sounds of frequencies lower than approximately 300 Hz (Ekimov and Sabatier 2006). The net force, F, exerted by the foot against the ground will produce a time-varying sound spectrum, in which the higher frequencies (in contrast to the GRF) depend on footwear and ground surface characteristics (Ekimov and Sabatier 2006).

Running is defined as a gait in which there is an aerial phase, a time when neither foot touches the ground. Walking has by definition no aerial phase. The stance of each foot is shorter in running, while the swing shows the opposite trend (Novacheck 1998). Pacing of barefoot running in athletes is usually greater than 170 SPM (GC < 0.35 s) (Lieberman 2012). Barefoot locomotion produces a greater disturbance than running when shod (Light et al. 1980). During barefoot running at 4 m/s on a hard surface, the magnitude of the peak of the GRF is between 1.5 and 2.5 body weight. This sends a shock wave up the body that can be measured in the head about 10 ms later (Lieberman et al. 2010). In theory that shock wave is likely to produce substantial sound due to bone conduction. Data of the magnitude, characteristics, and duration of sounds produced are scarcer for running than for walking. Since the body has no contact with the ground during the swing, the amplitude of air-conducted and bone-conducted sounds of locomotion is likely to be significantly lower in the swing phase. Due to a short stance and long swing period with no contact with the ground, the proportion of relatively silent periods is likely to be longer in running than in walking.

### Human sounds of ventilation

Data concerning non-human primate breathing sounds are not available, and in humans, data on the amplitude and bandwidth of respiratory sound at the ear canal are lacking. Inspiratory sound recorded outside of the mouth at a roughly average flow rate of 60 L/min has been shown to have a mean amplitude of 51 dB (Forgacs et al. 1971). The sound waves were of random amplitude with a regularly spread frequency distribution ranging from about 200 to 2,000 Hz. Groger and Wiegrebe (2006) reported that the external amplitude of human respiration sounds in non-exercise, calm nose breathing range from 25 to 35 dB. In unpublished experiments, I found that breathing of a human male instructed to maintain normal breathing volume, inspiring through the nose and expiring through the mouth at a frequency of 15 breaths per minute, increased the sound level by 34 dB LAeq (from 19 to 53 dB) approximately 5 cm from the ear (Table 1). These studies measured sound transmitted by air conduction. In addition, breathing will result in self-generated sound transmitted to the inner ear via the bones of the skull (Moore 2003, pp. 22–23), which is likely to contribute to their masking potential. In analogy with locomotion sound, self-generated ventilation sounds may have a potential to mask the analogous breathing sounds produced by a nearby individual. Self-generated ventilation sounds (the masker) will usually have higher amplitude than those of a second individual (the signal) since the former is produced nearer to the listener. In the simple trial (see Table 1), frequencies of ventilation sounds overlapped substantially with speech, indicating the potential to mask vocal communication. People typically cease breathing in hearing experiments when they are trying to perceive speech of very low amplitude (Parivash Ranjbahr, personal communication). The term “breathtaking” may indicate a tendency of humans to inhibit breathing in moments of fear or excitement, however, that has not been reported in the scientific literature.

## Respiratory–locomotor coupling

Breathing and locomotion are interrelated, and respiratory–locomotor coupling (RLC) is evident in all classes of vertebrates (Bramble and Carrier 1983; Funk et al. 1992); however, I have not found any data of RLC in non-human primates. The adaptive value of RLC is poorly understood. Energy saving has been suggested, although supporting evidence is lacking (Boggs 2001; Funk et al. 1997; Tytell and Alexander 2007). Human coupling of locomotion and breathing does not seem to result in energy gain or obvious mechanical benefits (Banzett et al. 1992; Bernasconi and Kohl 1993; Wilke et al. 1975). Human runners employ several phase-locked patterns (4:1, 3:1 2:1 1:1, 5:2, and 3:2), with 2:1 appearing to be most common (Bramble and Carrier 1983). Wilke et al. (1975) suggested that the entrainment of breathing and locomotory cycles in humans is an expression of the ease with which breathing becomes entrained to various rhythmic events. Breathing in humans can be subconsciously entrained to many kinds of rhythmic events, such as finger tapping, that have no mechanical link to the respiratory system (Haas et al. 1986). Banzett et al. (1992) concluded that coordination of breathing and stride in humans belongs to this class of coupling phenomena and has no obvious mechanical advantage.

## Reduced masking through RLC

The benefits of RLC may include enhanced hearing through concurrent noise production and silent intervals along with auditory grouping of self-produced noise. RLC is also likely to produce rhythmic and more predictable noise (Larsson 2012a). The amplitude of respiration is positively correlated to the flow rate (Forgacs et al. 1971); therefore, inspiratory sounds, as well as the amplitude of locomotion sounds, are likely to increase during exercise. This may produce enhanced benefits in situations when breathing and locomotion generate high-amplitude noise. This suggestion is supported by the fact that the tendency of humans to entrain respiration and locomotion is stronger in running than when walking (Bechbache and Duffin 1977), since running usually produces more noise.

## Synchronization of breathing

In resting humpback whales, synchronized breathing is commonly observed (Cynthia D’Vincent, personal communication). Surface diving dolphins are another example of synchronized breathing in animals (Larsson 2012a). An adaptive result of synchronization of self-produced noise, leading to extended silent periods, may be reduced masking (Larsson 2012a). Yawn contagion has been demonstrated in humans and several non-human animal species such as dogs (Madsen and Persson 2013) and chimpanzees (Massen et al. 2012). Contagious yawning has been suggested to lead to synchronization of behavior, and in chimpanzees, it is most apparent among males (Massen et al. 2012). In humans, auditory cues have been reported to be stronger than visual contagious yawn cues (Arnott et al. 2009). Social coherence has often been suggested as the function of synchronized yawning (Massen et al. 2012), while its influence on hearing perception of animal groups has scarcely been explored.

## Synchronization of body movements in primates

Oullier et al. (2008) evaluated phase synchrony by requiring pairs of humans facing each other to actively produce actions, while seeing, or not seeing similar actions being performed. Phase synchrony (unintentional in-phase coordinated behavior) emerged when they were exchanging visual information, whether or not they were explicitly instructed to coordinate with each other. However, rhythmic movement in humans is more robustly connected to acoustic than to visual cues (Repp and Penel 2004). Little is known about spontaneous synchronization in other species than humans. Nagasaka et al. (2013) examined spontaneous behavior synchronization in Japanese macaques. Synchronization was quantified by changes in button-pressing behavior while pairs of monkeys were facing each other. Participant-/partner-dependent synchronization was observed. Visual information from the partner induced a higher degree of synchronization than did auditory information (Nagasaka et al. 2013). Zarco et al. (2009) conducted a comparison of psychometric performance in humans and rhesus monkeys. The tasks involved tapping on a push button to measure the participants’ ability to produce accurate time intervals. Their results suggested that the species have a similar timing mechanism when passage of time needs to be quantified for a single interval. Overall, human subjects were more accurate than monkeys and showed less timing variability, especially during the self-pacing phase when multiple intervals were produced. The authors suggested that the internal timing machinery in macaques is not capable of producing multiple consecutive intervals. The typical human bias toward auditory as opposed to visual cues for the accurate execution of time intervals was not evident in rhesus monkeys.

## Synchronization of steps

Walking side by side, people often subconsciously synchronize steps, suggesting that the perception of one’s partner directly influences gait in the absence of conscious effort or intent (Nessler et al. 2009, 2012; Nessler and Gilliland 2010; van Ulzen et al. 2008; Zivotofsky and Hausdorff 2007). When two individuals stroll on neighboring treadmills, the walking pattern of both is substantially changed (Nessler et al. 2009, 2011a). Each person makes fine adjustments of the locomotion kinematics in order to resemble their partner’s behavior (Nessler et al. 2012). In paired walking, participants can be phase locked with a phase difference close to 0° (in phase), or they can be phase locked with a phase difference close to 180° (anti-phase) with walkers contacting the ground simultaneously with opposite-side feet (Nessler et al. 2012). The latter means that the right foot of one walker and left foot of the partner will reach the ground almost simultaneously. Leg length difference has been found to be significantly related to locking of step (Nessler and Gilliland 2009). Since the level of frequency locking did not significantly differ with varying visual and auditory information, the authors suggested that only a small amount of sensory information was sufficient to cause unintentional synchronization. Interviews following these experiments indicated that a small amount of sound was often detectable while wearing earplugs or sound-restricting earmuffs, and several participants indicated that they could feel mechanical vibrations resulting from their partner’s steps (Nessler and Gilliland 2009). Such sound and vibrations may have provided sensory information about the partner’s locomotion even in the experimental conditions with restricted visual or auditory information. In healthy individuals attempting to walk in time with a metronome at 120 beats per minute (BPM), the average pace was 119.52 ± 3.12 SPM, demonstrating a high degree of synchronization with rhythmic auditory sounds (Bilney et al. 2005).

No doubt similarity of the biomechanical characteristics of the individuals influences synchronization (Nessler et al. 2009, 2011b). However, selective regulation of treadmill velocity and inclination can lead to synchronization among persons with large differences in leg length and preferred pace that otherwise would not exhibit this kind of interaction (Nessler et al. 2011b). There are limits to this synchronization behavior (Nessler et al. 2009, 2012; van Ulzen et al. 2008). Synchronization between partners is often transient (Nessler et al. 2009; Zivotofsky and Hausdorff 2007). Pedestrian-induced lateral vibration of footbridges has been described (Fujino et al. 1993; Dallard et al. 2001). Typically, the walkers have no intention to march in step, but have naturally fallen into step with each other, apparently after the bridge begins to sway (Dallard et al. 2001). Dallard et al. (2001) suggest that people in a crowd also tend to synchronize with one another when there is no pavement motion, but that the probability of synchronization increases with increasing pavement motion amplitude. People have a stronger tendency to synchronize their steps to an oscillating bridge when it has a frequency close to their natural walking or running frequency. Thus, lateral deck movement encourages pedestrians to walk in step, and this step synchronization increases the human force and makes it resonate with the bridge deck (Fujino et al. 1993; Dallard et al. 2001). Data on synchronization in runners are lacking; however, my own observations of running couples of similar leg length suggest a clear tendency toward pacing. Synchronized locomotion in non-human primates seems not to have been reported, but in light of the irregularity of most primate locomotion, discussed above, it seems unlikely to be prominent.

## Why do humans tend to synchronize movements?

Social dynamics have been proposed to influence synchronization, and an individual’s movement pattern has been characterized as the result of interaction between her/his ideal movement pattern and that of nearby individuals (Issartel et al. 2007). Walking at speeds that differ from one’s preferred pace may result in increased energy expenditure, and it has been suggested that energy cost may play a role in unintentional entrainment, i.e., walkers may compromise on a cadence in light of metabolic energy consumption (Nessler and Gilliland 2009). McNeill (1995) suggested that synchronization of movements in a group is a potent way of creating and sustaining community and communication. Merker (2000) hypothesized a potentially confusing auditory effect based on the mimicry of a large animal or the possibility of frightening enemies when groups of ancestors walked in synchrony. Acoustic effects of synchronization have otherwise been little discussed.

## Silent periods

Synchronization of movements in animal groups, such as surface diving dolphin groups synchronizing splashdown, might reduce auditory masking problems through periods of relative silence (Larsson 2009, 2012a, b). Human groups walking or running out of step are likely to produce a roughly consistent amount of sound energy over the entire time span. Noisy phases of the GC will rarely overlap; thus, the total time of relatively silent periods will be reduced compared to walking in pace. For example, three similar-sized men running in phase will produce relatively little noise during the swing. During the relatively noisy stance period, the sound energy will be three times that of one man. However, this means that the perceived sound will increase less than 6 dB (If the footstep sound of one man has a level of 60 dB, two men will produce roughly 63 dB, four men 66 dB, and an intermediate value for three men).

## Predictable noise

The ability to segregate and identify sound sources in an auditory scene, for example a listener’s ability to group signal components into auditory objects and consequentially separate discrete sources from a complex mixture of sounds, is known as “auditory scene analysis” and onset time is suggested to be a useful grouping cue (Bregman 1990). Synchronization of human gait may improve the capacity to discriminate sound sources, since the onset time of the sounds of GC will coincide. In synchronized walking, one’s own and an accompanying person’s footsteps may be grouped together to form an auditory object, improving the brain’s ability to discriminate footsteps from other sound sources. Moreover, it is likely that two humans walking in pace on a consistent surface will be familiar with the sound patterns produced. Predictability of masking sounds may reduce backward masking (that caused by noise following the signal) due to a learning effect (Kidd and Feth 1982; Moore 2003, pp. 107–116), which in turn may favor speech perception. Human speech perception often takes place against a background of intense and irrelevant noise (Darwin 2008). However, familiarity with the noise seems to reduce its masking potential. Word identification has been shown to be better in the presence of familiar background music than in that of unfamiliar background music (Russo and Pichora-Fuller 2008). A masker’s rhythmic properties seem to influence speech perception. Ekström and Borg (2011) investigated the masking effect of a piano composition, played at 60, 120, or 180 BPM, on speech perception thresholds. All masking sounds were presented at an equivalent sound level (50 dBA). Low octave and fast tempo had the largest masking effect. The normal walking tempo of humans is close to 120 SPM. Two people walking in pace at this tempo will produce a regular rhythm of 120 while unpaced walking, for instance 110 BPM combined with 130 BPM, will produce a faster and more unpredictable rhythm.

## Steps in evolution?

Primitive hominids lived and moved around in small groups (Wilson 2011, pp. 57–105). The noise generated by the locomotion of two or more individuals can result in a complicated mix of footsteps, breathing, movements against vegetation, echoes, etc. The ability to perceive differences in pitch, rhythm, and harmonies, all of which are components of “musicality,” could help the brain to distinguish among sounds arising from discrete sources, and also help the individual to synchronize movements with the group. Endurance and an interest in listening might, for the same reasons, have been associated with survival advantages eventually resulting in adaptive selection for rhythmic and musical abilities and reinforcement of such abilities. Listening to music seems to stimulate release of dopamine in humans (Meyer-Bisch 2005) and other animals (Panksepp and Bernatzky 2002; Sutoo and Akiyama 2004). Aiding in discrimination of important signals has been discussed as a major function of dopamine (Durstewitz et al. 1999). Rhythmic group locomotion combined with attentive listening in nature may have resulted in reinforcement through dopamine release. To speculate further, a primarily survival-based behavior may eventually have attained similarities to dance and music, due to such reinforcement mechanisms. Since music may facilitate social cohesion, improve group effort, reduce conflict, facilitate perceptual and motor skill development, and improve transgenerational communication (Huron 2001), music-like behavior may at some stage have become incorporated into human culture. Changizi (2011) proposed that the human brain was well prepared to exploit incidental sounds of locomotion throughout cultural development.

## Similarities between human movement, breathing, and music

According to Changizi (2011), the most informative sounds of moving individuals are the basic building blocks of music. Four properties of moving individuals correspond directly to four fundamental ingredients of music: (1) the distance to the sound source (i.e., the moving individual) corresponds to loudness in music, (2) directionality influences pitch through the Doppler effect, (3) the moving individual’s speed corresponds to tempo in music, and (4) the moving individual’s gait pattern corresponds to the rhythm in music. He presents a list (pp. 191–195) with 42 potential similarities between music and human movement. To this list may be added that passive listening to music, or imagining it, activates areas of the brain associated with motor behavior (Chen et al. 2006; Janata and Grafton 2003). Listening to a rhythm often stimulates body movements (Grahn and Brett 2007). Rhythm information may be represented and retained in the brain as information about bodily movements (Konoike et al. 2012). Interactions between auditory and motor systems are important for the execution of rhythmic movements in humans, and music has a remarkable ability to drive rhythmic, metrically organized, motor behavior (Zatorre et al. 2007). Phillips-Silver and Trainor (2005) demonstrated a strong multisensory connection between body movements and auditory rhythm processing in infants. To tap or move in rhythm to music is rare during the first year of human life but steadily increases until the age of puberty (Drake 1997; Hugardt 2001; Merker 2005), a timetable that shows some analogies with the child’s increasing capacity to walk. Music often influences emotions and vice versa. Interactions between locomotion sound and emotions have also been demonstrated. Giordano and Bresin (2006) suggested that locomotion sounds may be influenced by the emotion of the walker, and according to Bresin et al. (2010), the sounds produced on a more firm surface lead to more aggressive walking patterns. Runners changed step length and thereby the speed when music of different “emotional” character was recorded, although the pace was the same in all conditions (130 BPM) (Leman et al. 2013). Walking and running will usually produce rhythms in the range of 75–190 BPM. People can synchronize walking movements with music over a broad spectrum of tempos, but this synchronization is optimal in a narrow range around 120 BPM (Styns et al. 2007). Music is often played at a tempo similar to walking (Changizi 2011, p. 191). Respiration frequency can be increased by musical stimuli, and the increased breathing rate secondarily increases heart rate and blood pressure. This increase has been shown to be proportional to the tempo of music (Bernardi et al. 2006). A slow tempo (60–80 beats per minute) is related to relaxation and pain relief. Silence (a pause from music) further increases relaxation (Bernardi et al. 2006). The phase-locked patterns in human runner and walker RLC, 4:1, 3:1, 2:1, contribute to similarities between locomotion/ventilation sounds and rhythms in music.

## Discussion and conclusions

Human locomotion and ventilation noise seem to have the potential to mask critical sounds in the environment, such as the footsteps and breathing of a stalker or prey. Synchronized walking of people in small groups is likely to reduce the masking properties of locomotion sounds. Possible adaptive advantages could be early detection of stalkers and enhanced perception of vocal communication within the group. Thus, the acoustic advantages that have been suggested for schooling fish, dolphin, and bird groups (Larsson 2009, 2012a, b) may also be relevant for humans moving in synchrony. Moreover, limited data suggest that locomotion sounds may be used subconsciously to achieve synchronization of group locomotion (Nessler and Gilliland 2009; Fujino et al. 1993; Dallard et al. 2001). Changizi (2011) suggests that the brain became “harnessed by music,” proposing that the fundamental ingredients of music developed to be similar to the sounds produced by a moving individual, since the human brain was adept at interpreting and analyzing such sounds in nature. The evidence presented here suggests an evolutionary adaptation of the auditory system to perceive and analyze rhythmic locomotion sound, complementing Changizi’s premise.

Archaeological data indicate that, in primitive societies, anywhere from 10 to 60 % of men died by homicide or in warfare (Bowles 2009). Thus, abilities to reduce masking and increase the chance of hearing an approaching enemy would have had high adaptive value in bipedal hominids. However, reducing masking from incidental sounds of locomotion is as likely to have adaptive value in non-human primates. Groups of arboreal primates would also benefit from simultaneous movement and pauses to produce silent intervals. The spontaneous behavior synchronization demonstrated among Japanese macaques (Nagasaka et al. 2013) may offer such acoustic benefits in nature. The higher degree of synchronization induced by visual information from the partner, as opposed to auditory cues, does not preclude a contribution of reduced auditory masking. Since auditory cues created by their locomotion are less repetitive and predictable than human steps, tree-climbers’ visual cues to synchronize locomotion may be more reliable than auditory cues. This may also provide a rationale for the non-human primates’ inability to accurately produce multiple tap intervals (Zarco et al. 2009), and possibly explain why monkeys detect rhythmic groups in music, but not the beat (Honing et al. 2012). Beat induction is the cognitive skill that let us pay attention to a regular pulse in music to which we can then synchronize. Perceiving this regularity in music allows humans to dance and create music together. Beat induction is a fundamental musical characteristic that, possibly, played a crucial role in the origins of music (Honing 2012). It is clearly correlated with motor activities, and increasing evidence shows that the neural circuits involved in beat perception overlap with motor circuitry even in the absence of overt movement (McAuley et al. 2012). Successful beat induction was diminished when the implied beat was at a slower cadence (1,500 ms or 40 BPM) compared with a quicker tempo (600 ms or 100 BPM) (McAuley et al. 2012) that corresponds to a normal human gait tempo.

The lack of empirical research on locomotion and ventilation sounds is a major limitation and should be an incentive for further research, e.g., about the prevalence of human walking in step and the neurophysiological mechanisms behind. The list of further research topics could also include perceptual factors such as acoustics (background noise, hearing acuity, level of sound generated) and the role of vision in synchronization of steps; how pacing influences vocal communication and vice versa; the masking potential of locomotion and ventilation sounds, not least the masking potential of different phases of the GC and the respiratory cycle; masking due to bone-conducted locomotion and ventilation sounds; the possible suppression effect of self-generated locomotion/ventilation sounds in the CNS; acoustic consequences of RLC in vertebrates; and comparative analyzes of acoustic and rhythmic properties of human bipedal walking versus arboreal locomotion and quadruped walking in apes.

Most, if not all, vertebrates are capable of auditory learning, which essentially means an ability to make associations with sounds heard, but few are capable of vocal learning, the ability to modify acoustic and/or syntactic structure of sounds produced, including imitation and improvisation (Jarvis 2007). Vocal learning has been found in humans, bats, cetaceans, pinnipeds, elephants, parrots, hummingbirds, songbirds (Jarvis 2007), and recently also in the ultrasound register of mice (Arriaga et al. 2012). The vocal learning and rhythmic synchronization hypothesis proposes that vocal learning provides a neurobiological foundation for auditory/motor entrainment (Patel 2006). Schachner et al. (2009) suggested that entrainment to auditory beats emerged as a by-product of the capacity for vocal mimicry. Spontaneous motor entrainment to music has been demonstrated in vocal learners such as parrot and elephant species (Patel et al. 2009; Schachner et al. 2009). However, entrainment has recently been demonstrated in the less vocally flexible California sea lion, which has been suggested to be a limitation of the vocal learning and rhythmic synchronization hypothesis (Cook et al. 2013). This review article suggests the alternative view: that repetitive and predictable locomotion sounds influenced the development of entrainment in humans. It is likely that animal species that display oscillating, predictable locomotion patterns also produce rhythmic and predictable sounds of locomotion. An interesting question for the future is whether exposure to repetitive sounds of locomotion may stimulate the evolution toward auditory–motor entrainment. A related question is whether auditory–motor entrainment may stimulate the evolution of vocal abilities. Several vocal learning species produce oscillating movement patterns for long periods when they are moving in groups, for example human and elephant gait; coast and burst swimming in cetaceans and pinnipeds; wing flapping in bats, parrots, hummingbirds, and not least swarms of songbirds. At least bats and birds use their forelimbs to a large extent during locomotion. Brain structures involved in vocal communication in vertebrates are closely linked to motor processing of the forelimbs (Bass and Chagnaud 2012). Developmental studies of sound-producing fishes and tetrapods reveal that structures in the nervous system dedicated to vocalization originate from the same caudal hindbrain rhombomere (rh) 8-spinal compartment (Bass and Chagnaud 2012). Midshipman fish and the hitherto investigated tetrapods have forelimb motoneurons that function in both sonic and gestural signaling, and vocal and pectoral systems seem to have a shared developmental origin. In addition, vocal and pectoral systems have been proposed to possess shared social signal functions (Bass and Chagnaud 2012). Although the hypothesis presented here proposes a connection of music with motor processing of the hind limbs, a high degree of neuronal coordination of arm and leg movements has been demonstrated during human locomotion (Dietz et al. 2001).

Studies of interactions between movements and sound perception may increase the understanding of synchronized flock behavior in animals, including humans. Human synchrony phenomena related to walking, its acoustic and social significance, and the brain processes involved are little understood and may provide interesting areas for future research, not least bipedal walking and the evolution of rhythmic and musical abilities.

## Footnotes

1 Decibel (dB) is a logarithmic scale, which means that doubling the sound energy will increase the dB value by 3 dB. Equivalent continuous level or LAeq (A = average) is equivalent to the level of continuous noise given in decibels A (dBA) and integrates sound energy measured over a period of time (approximately 10 s in these recordings) to adjust for fluctuation of usual noise levels. The dBA filter is widely used. dBA roughly corresponds to the inverse of the 40 dB (at 1 kHz) equal-loudness curve for the human ear; using the dBA filter, the sound level meter is less sensitive to very high and very low frequencies. Measurements made with this scale are expressed as dBA (Meyer-Bisch 2005). These recordings (using LAeq) did not demonstrate differences in amplitude between relatively silent and relatively noisy phases of the gait cycle.
